# Supplementary material for: Content-rich biological network constructed by mining PubMed abstracts
Source: BMC Bioinformatics. 2004 Oct 8;5:147. doi: 10.1186/1471-2105-5-147 (PMC528731; doi:10.1186/1471-2105-5-147)
Supplement: Additional File 2 — The original results of the above study (non-essential files are deleted to keep the file size under the limit set by BMC bioinformatics). [file 1471-2105-5-147-S2.bz2 › chilibotAdditionalFile2/dip50/155ID8822197E554/html/RB1_CDK6.html]

 


 **RB1** and **CDK6** 
  
Found 151 abstracts in PubMed, retrieved 50.  
 

 What does Google say? 
 PDF only 
| .edu only 

---

**Interactive relationship** (e.g. stimulation, inhibition, etc)

**Inhibitory relationship**- Mitogen dependent, cyclin D dependent kinases cdk4 and  **cdk6**  phosphorylate the retinoblastoma  [ **RB1** ]  Rb tumor suppressor protein, helping to cancel its growth inhibitory effects and enabling E2F transcription factors to activate genes required for entry into the DNA synthetic phase S of the cell division cycle.  Ref: 10919634 Cancer Res, 2000
- Most commonly, such mutations involve CDKN2A, a cyclin dependent kinase inhibitor of two kinases, CDK4 and  **CDK6** , which phosphorylate the retinoblastoma  [ **RB1** ]  protein pRB and thereby promote passage through the G1 S cell cycle restriction point.  Ref: 10777219 Oncogene, 2000
- The consequent induction of immediate early response genes, such as cyclins, and subsequent activation of cyclin dependent kinases CDK4,  **CDK6**  and CDK2 inactivates the retinoblastoma  [ **RB1** ]  family of proteins pRb, p107 and p130, together termed pocket proteins, and releases their suppressive association with E2F transcription factors.  Ref: 10761990 Pigment Cell Res, 2000
- DIF 1 inhibited the phosphorylation of the retinoblastoma  [ **RB1** ]  protein and the activities of cyclin dependent kinase Cdk 4,  **Cdk6** , and Cdk2, which phosphorylate the retinoblastoma  [ **RB1** ]  protein.  Ref: 10625307 Circ Res< MedlineTA>Circ Res, 2000
- At the molecular level, the tetramer inhibited cyclin dependent kinase CDK 6  [ **CDK6** ]  expression and activity, induced an increase in the level of p27 kip1, and reduced the level of retinoblastoma  [ **RB1** ]  protein expression.  Ref: 12874002 Cancer ResCancer Res, 2000
**Neutral relationship**- In human T lymphocytes  **cdk6**  has been shown to be the initial retinoblastoma  [ **RB1** ]  protein kinase detectable at mid G1.  Ref: 11804329 Mol Cells, 2001
- The phosphorylation of retinoblastoma  [ **RB1** ]  protein from T cells was detected very early in G1 and was associated mainly with  **cdk6**  cyclin D2 complexes which accounted for a minor portion of the total cellular  **cdk6**  contained in the cytoplasmic fraction of T cells and mostly in the catalytically inactive form.  Ref: 11804329 Mol Cells, 2001
- D type cyclins bind to and activate the cyclin dependent kinases Cdk4 and  **Cdk6** , which in turn phosphorylate their downstream target, the retinoblastoma  [ **RB1** ]  protein Rb.  Ref: 12459251 Gene, 2002
- CDK4 and  **CDK6** , which are activated by D type cyclins during the G 1 phase of the cell cycle, are thought to be responsible for phosphorylation of the retinoblastoma  [ **RB1** ]  gene product pRb.  Ref: 11003668 Mol Cell Biol, 2000

**Non-interactive relationship** (e.g. studied together, co-existance, homology, etc.)

- Thus, CAK phosphorylated K cyclin  **cdk6**  targets multiple sites in the retinoblastoma  [ **RB1** ]  protein pRb whereas the unphosphorylated complex targets a single site.  Ref: 11423981 Oncogene, 2001
- p16 regulates the G 1 S cell cycle transition by inhibiting the cyclin D cyclin dependent kinase CDK 4  **CDK6**  mediated phosphorylation of retinoblastoma  [ **RB1** ]  protein pRb.  Ref: 11705866 Clin Cancer Res, 2001
- The functional characteristics of ICAM 1 osteoblasts were emphasized further by cell cycle regulation, as manifested by i up regulation of p53 and p21, ii reduction of activity of cyclin dependent kinase cdk 6  [ **CDK6** ] , iii underphosphorylation of retinoblastoma  [ **RB1** ]  protein, iv increased Fas but reduced bcl 2 expression, and v majority of cells remained at G0 G1 phase.  Ref: 11028443 J Bone Miner Res, 2000
- This G 1 arrest is associated with inhibition of cyclin D3  **cdk6**  mediated retinoblastoma  [ **RB1** ]  protein phosphorylation by p18 INK4c.  Ref: 11035070 J Immunol, 2000
- Components upstream of the retinoblastoma  [ **RB1** ]  tumour suppressor pRB, namely the INK4 family of cyclin dependent kinase CDK inhibitors, the D type cyclins, their partner kinases CDK4  **CDK6** , and pRB as their critical substrate, are differentially targeted in diverse types of cancer.  Ref: 12754735 J Pathol< MedlineTA>J Pathol, 2000
- In HepG2 cells, this inhibition was associated with an arrest of the cell cycle in G G, increased cellular levels of p21, decreased levels of the hyperphosphorylated form of the retinoblastoma  [ **RB1** ]  protein, and decreased levels of cyclin D1, but no significant changes were seen in the levels of the p16, p27, cyclin dependent kinase 4, cyclin dependent kinase 6  [ **CDK6** ] , glycogen synthase kinase 3beta, or beta catenin proteins.  Ref: 12124333 Cancer Res, 2002
- Furthermore, BMP 7 caused an upregulation of the cyclin dependent kinase inhibitors CDKIs p21 and p27, decreased activity of Cdk2 and  **Cdk6** , and hypophosphorylation of the retinoblastoma  [ **RB1** ]  protein pRb.  Ref: 11453659 Biochem Biophys Res Commun, 2001
- Progression through the G1 phase of the cell cycle requires phosphorylation of the retinoblastoma  [ **RB1** ]  gene product pRb by the cyclin D dependent kinases CDK4 and  **CDK6** , whose activity can specifically be blocked by the CDK inhibitor p16 INK4A.  Ref: 11278443 J Biol Chem, 2001
- Despite the significant amounts of CDK4 and  **CDK6** , only nonphosphorylated forms of retinoblastoma  [ **RB1** ]  protein were detectable in fully luteinized cells.  Ref: 10775192 Biol Reprod, 2000
- Abrogation of mitogenic and anti mitogenic response regulatory proteins, such as the retinoblastoma  [ **RB1** ]  tumor suppressor protein, cyclin D1, cyclin dependent kinase 6  [ **CDK6** ] , and CDK inhibitors, p27, and p16, occur frequently in human oral cancers.  Ref: 12097237 Crit Rev Oral Biol Med, 2002
